# Application of Ammonium Persulfate for Selective Oxidation of Guanines for Nucleic Acid Sequencing

**DOI:** 10.3390/molecules22071222

**Published:** 2017-07-21

**Authors:** Yafen Wang, Chaoxing Liu, Tingting Hong, Fan Wu, Shuyi Yu, Zhiyong He, Wuxiang Mao, Xiang Zhou

**Affiliations:** 1 Key Laboratory of Biomedical Polymers of Ministry of Education, College of Chemistry and Molecular Sciences, Wuhan University, Wuhan 430072, Hubei, China; whuwangyafen@126.com (Y.W.); chaoxingliu@whu.edu.cn (C.L.); htting2006@126.com (T.H.); hanyezhuhuo@whu.edu.cn (F.W.); 15827423361@163.com (S.Y.); hezy@whu.edu.cn (Z.H.); 2 The Institute for Advanced Studies, Wuhan University, Wuhan 430072, Hubei, China; 3 Hubei Collaborative Innovation Center for Green Transformation of Bio-Resources, College of Life Sciences, Hubei University, Wuhan 430062, Hubei, China; maowuxiang@163.com

**Keywords:** guanine, ammonium persulfate, nucleic acid

## Abstract

Nucleic acids can be sequenced by a chemical procedure that partially damages the nucleotide positions at their base repetition. Many methods have been reported for the selective recognition of guanine. The accurate identification of guanine in both single and double regions of DNA and RNA remains a challenging task. Herein, we present a new, non-toxic and simple method for the selective recognition of guanine in both DNA and RNA sequences via ammonium persulfate modification. This strategy can be further successfully applied to the detection of 5-methylcytosine by using PCR.

## 1. Introduction

Nucleic acids, which are considered the most fundamental component and essential biological macromolecules of life, play a vital role in many cellular processes. A precise sequencing of nucleic acids is essential for understanding living matter [1,2]. For this purpose, selective cleavages at specific bases in DNA and RNA provide an effective approach to identifying the position of each base in nucleic acids. Various biological, chemical and physical factors can give rise to DNA damage and induce cleavage [3,4,5,6,7,8], particularly in vulnerable guanines, which are most likely to undergo oxidation due to their electron-rich properties. To date, many efforts have been devoted to developing biological and chemical methods for the selective cleavage of guanines in nucleic acids.

A number of metals (Cu^II^, Ni^II^, Mn^II^) and metal ligands (trinuclear copper complex, porphyrins and corroles) have been explored to damage guanine in aqueous solution via oxidation. Using these compounds, different guanines in diverse structures can be recognized. For instance, Mn-corroles can transfer active oxygen into the guanine of a non-duplex region in DNA [5], and trinuclear copper complexes can selectively recognize guanines of double helix-coil junctions in DNA [9]. The orientation of each individual G-tract in the G-quadruplex in single and double stranded nucleic acids were dissected by ligand induced photocleavage footprinting [10]. Using suprofen, G-specific sequencing can be realized by UV [11]. Assisting with light and oxygen, methylene blue is sensitized for the photooxidation of guanine [12,13]. Further, Ribonuclease T1 (RNase T1) from aspergillus oryzae is an endoribonuclease that can hydrolyse the residues following guanine in single-stranded regions of RNA [14]. Due to its specificity for guanine, RNase T1 is widely used to digest denatured RNA prior to sequencing [15]. The most classical chemical sequencing proposed by Maxam and Gilbert relies on the base-specific damage of DNA followed by hot piperidine to cleave the damaged sites [16]. In this sequencing system, guanines can be alkylated by dimethyl sulphate (DMS) and stopped by mercaptoethanol, followed by hot piperidine to cleave the guanine residues. Our group recently developed a method for the selective recognition of guanine in DNA/RNA non-duplex regions through potassium tungstate and hydrogen peroxide treatment [17]. Although these methods have provided vital information for the G-ladder in nucleic acids, some deficiencies still exist that need to be overcome before this can be made a convenient and safe way to produce successful G sequencing. For example, the synthesis process of metal complexes is cumbersome; RNase T1 is very expensive; DMS is highly toxic, which can lead to chromosomal alterations; and ethanethiol is effluvial and poisonous. Accordingly, developing a convenient and facile approach to the G-ladder is of great significance.

**Figure 1:**
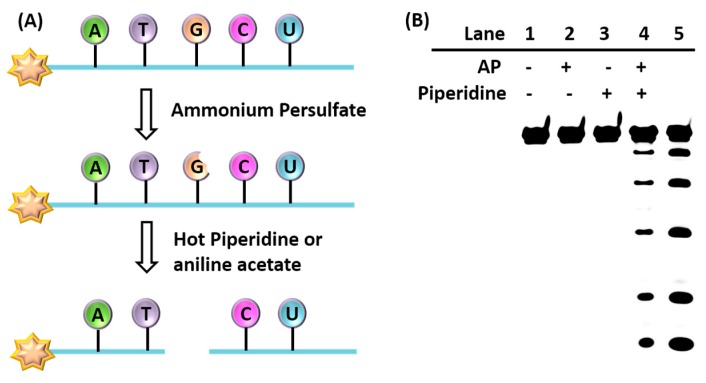
(**A**) Schematic illustration of nucleic acid sequences oxidized by AP and then cleaved by hot piperidine or aniline acetate; (**B**) Polyacrylamide gel electrophoresis experiments showing cleavage products of HEX-labeled DNA of ODN-mismatch (20 pmol) incubated with AP in Tris-HCl buffer (pH = 7.4). Lane 1: DNA alone; lane 2: ODN with AP; lane 3: ODN with piperidine; lane 4: ODN with AP and piperidine; lane 5: G-ladder of ODN (treated with DMS).

Herein, we have developed a less-toxic and low-cost way to obtain the G-ladder in various DNA and RNA structures. The principle that we designed for selective oxidation of guanine is illustrated in Figure 1A, in which the hexachlorofluorescein (HEX)-labelled DNA sequence was treated with ammonium persulfate (AP). Then, the guanine in the nucleic acid was oxidized, followed by treatment with hot piperidine to cleave the residue site. The condition of the RNA cleavage reaction was slightly different and was cleaved by aniline acetate buffer (pH 4.5). After that, the purified product was analyzed by denaturing polyacrylamide gel electrophoresis (PAGE).

## 2. Results

### 2.1. Single-Strand Oxidation by AP

First, to investigate which compound or mixture could selectively damage guanine and further induce the cleavage of guanine residues in DNA sequences, PAGE analysis was performed (Figure 1B). As mentioned above, an obvious selective cleavage of guanine was only observed when the HEX-labelled DNA was incubated with AP and then treated with hot piperidine (lane 4). Without hot piperidine, no new bands appeared on the PAGE (lane 2). The same result was observed in the absence of AP (lane 3). Taken together, after treatment with AP, the nucleic acid was damaged at guanine sites to produce residues, followed by treatment with hot piperidine to cleave those residues and obtain new bands in the PAGE. This allowed us to determine the guanine content in the analyzed sequence. To obtain better results for the loci of guanines in the tested nucleic acid sequences, the AP concentration and the reaction time were further studied (Supplementary Figures S1 and S2). From the results, 10 mM AP and the time of the reaction was 30 min were chosen for further study.

### 2.2. Oxidation of Guanines with Diverse DNA Structures by AP

**Figure 2:**
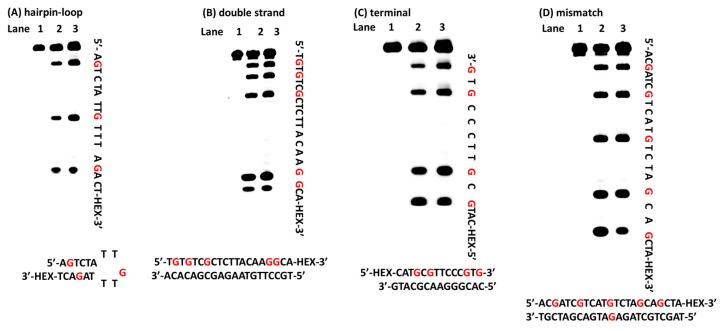

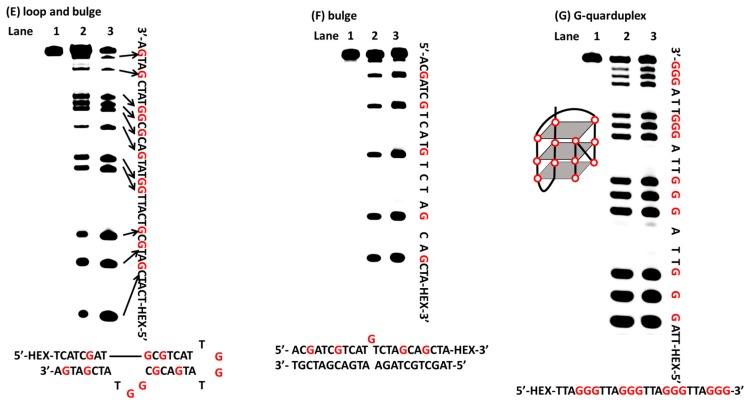
Polyacrylamide gel electrophoresis analysis of DNA. The oxidized DNA treated with piperidine at 90 °C for 40 min. (**A**) Cleavage of the hairpin-loop structure; (**B**) Cleavage of the double strand structure; (**C**) Cleavage of the terminal structure; (**D**) Cleavage of the mismatch structure; (**E**) Cleavage of the loop and bulge structure; (**F**) Cleavage of the bulge structure; (**G**) Cleavage of the G-quadruplex structure.

**Figure 3:**
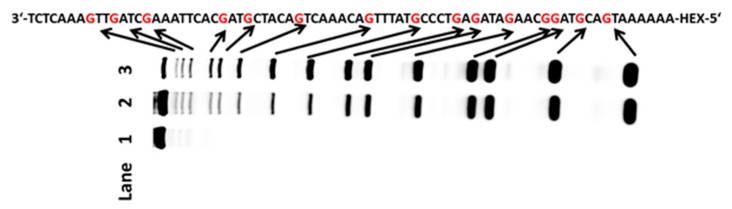
Polyacrylamide gel electrophoresis analysis of 76-mer DNA. The ODN (20 pmol) was oxidized by AP and then treated with piperidine. Lane 1: DNA alone; lane 2: DNA treated with AP; lane 3: G-ladder of ODN (treated with DMS).

As is well known, there are many different structures in nucleic acids; therefore, it was important to determine whether the approach that we explored could selectively detect guanine in any structure. First, a DNA sequence containing a hairpin-loop was chosen for testing. The tested sequence was incubated with AP in 37 °C for 30 min. After desalting, the oxidized DNA was treated with hot piperidine. Finally, the products were fractionated by PAGE. The new bands (lane 2 in Figure 2A), which were consistent with the bands in lane 3 (ODN was treated with DMS), indicated the guanines in the ODN-hairpin-loop (Figure 2A). In addition to the hairpin-loop, DNA sequences containing loop-bulge and bulge structures were applied in our system. PAGE analysis indicated that all of the guanines in the tested ODNs could be recognized (lane 2 in Figure 2E,F). Due to the frequent occurrence of mismatches during the process of DNA replication, it was also important that this approach could oxidize guanine in a mismatched sequence; therefore, a DNA sequence containing mismatches was tested. The new bands on the PAGE matched the guanines in the mismatched DNA sequence (lane 2 in Figure 2D). In addition, double strand and terminal DNA are common structures in nucleic acids. These structures were chosen for further experimentation. New bands observed in lane 2 identified the guanines of the tested sequences (lane 2 in Figure 2B,C). The widespread nature of G-quadruplex forming sequences in genomic DNA and their role in regulating gene expression means that G-quadruplex structures play a vital role in life process. The bands reflecting cleavage in the gel indicated that the loci of the guanines in the tested G-quadruplex could be completely recognized (lane 2 in Figure 2G). To know whether different dyes and labelling positions would affect the result, other nucleic acids, such as FAM labelled nucleic acids (Supplementary Figures S6 and S7) were also tested to examine the feasibility of AP for G-sequencing. We used CD to confirm the structures of G-quadruplex and G-triplex (Supplementary Figures S9–S11). To evaluate whether the method that we established could be applied to a longer fragment, a 76-mer HEX-labeled DNA was chosen. As shown in Figure 3, the new bands in lane 2 indicated the guanines in the tested sequence. Therefore, the system that we developed could recognize guanines in both single- and double-stranded DNA, and could identify guanines in longer sequences. Furthermore, different dyes (HEX or FAM) and labelling positions (5-terminal or 3-terminal) could not affect the reaction between ammonium persulfate and nucleic acids.

### 2.3. Oxidation of RNA Sequences by AP

**Figure 4:**
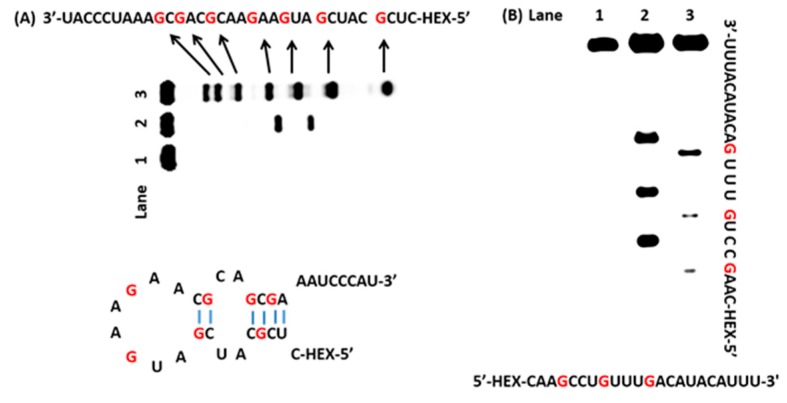
Polyacrylamide gel electrophoresis analysis of RNA sequences treated with AP and aniline acetate. (**A**) Lane 1: RNA alone; lane 2: RNA was treated with RNase T1; lane 3: RNA was treated with AP and aniline acetate; (**B**) Lane 1: RNA alone; lane 2: RNA was treated with AP and aniline acetate; lane 3: RNA was treated with RNase T1.

The application of the developed system to RNA was also vital; therefore, we tested our method with RNA molecules. The RNA sequences were oxidized by AP, followed by treatment with aniline acetate (pH 4.5). As expected, this system produced cleavage bands at the sites of guanines in both single- and double-stranded RNA (lane 3 in Figure 4A and lane 2 in Figure 4B). RNase T1 is a fungal endonuclease that hydrolyzes residues following guanine to produce a 3′-phosphate, the migration rate of RNase T1 digestion is faster than that of aniline acetate treatment, which led to the formation of a 3′-protonated Schiff base that retarded migration [18]. We compared the new bands, which were produced by AP/aniline acetate (lane 3 in Figure 4A), with RNase T1, which could only recognize guanine in single-strands (lane 2 in Figure 4A). The ability of AP to recognize guanine in RNA is superior to RNase T1, which is unable to recognize 3′-terminal guanine because RNase T1 cleaves the 3′-phosphate group of ribonucleotide in guanine and the 5′-hydroxyl of the adjacent nucleotide. The results confirmed that guanines in single and double regions of RNA can be oxidized by AP, and that the residues can be cleaved by aniline acetate. Based on these observations, the system that we developed was able to recognize guanine in both single-and double-stranded DNA or RNA.

### 2.4. AP for G-Sequencing Compared with Methylene Blue for G-Sequencing

**Figure 5:**
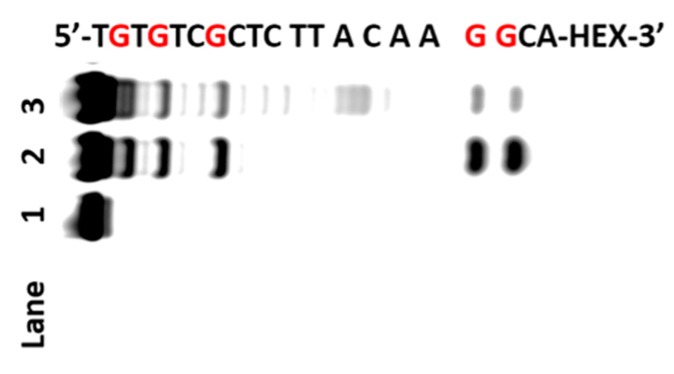
Polyacrylamide gel electrophoresis analysis of 20 mer-DNA. Lane 1: DNA alone; Lane 2: DNA was treated with AP; Lane 3: DNA was treated with methylene blue in the presence of light and oxygen for 15 min.

Photo-oxidation reagent-methylene blue can be used as a base-specific DNA sequencing reagent for G-sequencing. The method we established was also compared with methylene blue. We chose a single-stranded DNA molecule for the experiment. As shown in Figure 5, new bands appeared following PAGE analysis of the guanines sites in the tested oligonucleotide. Thus, methylene blue photo-oxidization is not selective for guanine.

### 2.5. Detection of 5-Methylcytosine Based on PCR and AP

**Figure 6:**
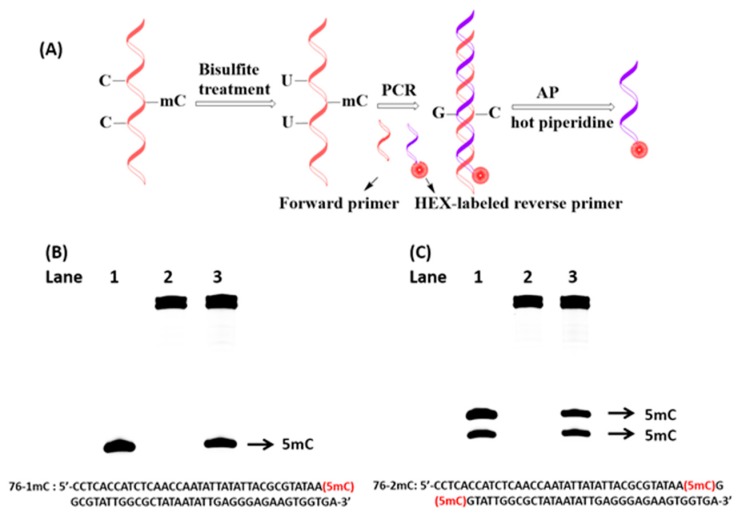
(**A**) Schematic illustration of detection of the loci of 5-methylcytosine in DNA by PCR, the product of PCR was treated with AP and hot piperidine; (**B**,**C**) Poly-acrylamide gel electrophoresis analysis of 76-mer template containing one or two 5-methylcytosine sites. Lane 1: marker; lane 2: PCR product without treatment; lane 3: PCR product treated with AP and hot piperidine.

Cytosine has many natural modifications, such as 5-methylcytosine, 5-hydroxycytosine and further oxidation products [19]. 5-methylcytosine is an essential epigenetic modification that plays a vital role in the regulation of gene expression. Aberrant DNA methylation has been found to be associated with many diseases, especially cancers. Because the system that we established could recognize guanine in longer strands, a strategy was designed to detect 5-methylcytosine, as shown in Figure 6A. First, the DNA sequence was treated with bisulfite such that C would be oxidized to uridine (U) with a negligible influence on 5-methylcytosine [20]. Then, the sample was subjected to bisulfite treatment and used as the template for polymerase chain reaction (PCR). Because only 5-methylcytosine in the original DNA remains intact after bisulfite treatment, and it is matched to guanine (G) in the complementary DNA strand, the presence of 5-methylcytosine can be detected by the presence of G on the complementary DNA strand. Because AP has a high specificity for recognizing G, we attempted to use this system to detect 5-methylcytosine by PCR product. One or two new bands appeared in gels, which were consistent with the site of 5-methylcytosine that we tested previously (Figure 6B,C). Based on these results, the system that we established can be used to detect 5-methylcytosine based on PCR.

### 2.6. LC-MS Analysis of Guanine Oxidized by AP

8-Oxo-7,8-dihydro-2′-deoxyguanine (8-oxoG) is one of the biomarkers in DNA oxidative damage [21], and plays a major role in mutagenesis, aging and carcinogenesis [22]. 8-oxoG is a problematic lesion to detect in nucleic acid [23], and could be further oxidized to the labile products-dGh [24], which are sensitive to hot piperidine. We predict that G can be oxidized into 8-oxoG and further formed into a labile product by treatment with AP. To verify the oxidized product of guanine by AP, we used a liquid chromatograph-mass spectrometer (LC-MS) to analyze the digested nucleosides of an oligodeoxynucleotide that had been treated with AP. By extracting ions, the mass theoretical value of dGh is 274.11460, and we obtained a value of 274.11370 (Figure S13). Thus, our results suggest that AP could oxidize guanine to 8-oxo-dG, and could be further oxidized to dGh.

## 3. Materials and Methods

### 3.1. Chemicals and Materials

All chemicals were purchased from Beijing Innochem Sci. & Tech. Co. Ltd. (Beijing, China) unless mentioned otherwise. The DNA oligonucleotides labeled with hexachlorofluorescein (HEX) or 6-carboxy-fluorescein (FAM) were purchased from GeneCreate Co., Ltd. (Wuhan, China). The HEX-labeled RNA were purchased from Takara Biotechnology (Dalian, China). LC-MS data was collected with the Agilent™ 1220 Infinity LC combined with the 6120 Single Quadrupole mass spectrometer (Agilent Technologies, Santa Clara, CA, USA). Brucker Daltonics (Manning Park Billerica, MA, USA) APE XII 47e via using an electrospray ionization (ESI)-positive mode. Circular dichroism (CD) experiments utilized a Jasco-810 spectropolarimeter (Jasco, Easton, MD, USA). DNA concentration was quantified by NanoDrop 2000c (Thermo Scientific, Waltham, MA, USA). Ammonium persulfate (AP) was purchased from Sinopharm Chemical Reagent Co., Ltd. (Shanghai, China). All water used in this study was ultrapure water (18.2 MΩ/cm).

### 3.2. Preparation of DNA Containing Different Structures

DNA sequences containing different structures were annealed under the condition of 10 mM Tris-ethylenediaminetetraacetic acid (EDTA) (pH 7.4) and 100 mM NaCl by heating 95 °C for 5 min then slowly cooling to room temperature to form the desired structures. G-quadruplex and G-triplex DNA were prepared in 10 mM Tris-HCl buffer (pH 7.4) and 100 mM KCl by heating DNA to 95 °C for 5 min and then gradually cooled to room temperature at a constant rate over the course of 1.5 h to form G-quadruplex or G-triplex. To determine RNA sequences, the molecules were annealed under the conditions of 10 mM MgCl_2_, 100 mM NaCl and 10 mM potassium phosphate (pH 7.4) by heating 65 °C for 5 min then slowly cooling to room temperature over the course of 45 min to form the structures produced.

### 3.3. Circular Dichroism Studies

The conformation of G-quadruplex and G-triplex was examined by circular dichroism (CD). Spectra were measured at room temperature using a quartz cell with a 1 cm path length; CD spectra were collected from 220 nm to 320 nm and with a scanning speed of 200 nm/min. The bandwidth was 5 nm, and the response time was 2 s.

### 3.4. Cleavage of DNA/RNA

HEX or FAM labeled DNA sequence 2 µL (10 µM) was incubated with 2 µL Tris-HCl buffer (100 mM, pH 7.4), 2 µL AP (100 mM) and 14 µL H_2_O at 37 °C for 30 min. Then immediately transferred into 1.5 mL centrifuge tube. After that, 1 mL prechilled 100% ethanol and 10 µL 3 M Na-Acetate (pH 5.2) were added to the reaction tube and vortexing. The mixture was frozen at −80 °C for 2 h and centrifuged at 4 °C (12,000× *g*) for 20 min. After removal of the upper solution, the DNA precipitate was vacuum dried, then re-dissolved in 100 µL piperidine (*v*/*v* 10%) and heated at 90 °C for 40 min. After the piperidine treatment, the DNA was precipitated again as the step mentioned above. After being dried by vacuum, the DNA precipitate was dissolved in 80% formamide deionized for further examination on polyacrylamide gels.

HEX labeled RNA sequence 2 µL (10 µM) was incubated with 2 µL Tris-HCl buffer (100 mM, pH 7.4), 2 µL AP (100 mM) and 14 µL RNase free H_2_O at 37 °C for 5 min. Then immediately transferred into 1.5 mL centrifuge tube. After that, 1 mL prechilled 100% ethanol and 10 µL 3 M Na-Acetate (pH 5.2) were added into the reaction tube and vortexing. The mixture was frozen at −80 °C for 2 h and centrifuged at 4 °C (12,000× *g*) for 20 min. After removal of the upper solution, the RNA precipitate was vacuum dried, then re-dissolved in 20 µL aniline acetate (1 M, pH 4.5) and heated at 60 °C for 20 min in dark. After that, the DNA was precipitated again as the step mentioned above. After being dried by vacuum, the DNA precipitate was dissolved in 80% formamide, deionized for further examination on polyacrylamide gel electrophoresis (PAGE).

### 3.5. Primer Design and PCR Amplification

In order to achieve a successful PCR product, no CpG sites can exist in the primers for PCR. The PCR mixture contain 5 µL reaction buffer (10×), 4 µL dNTPs (2.5 mM), 2.5 units hot start Taq polymerase, 2 µL forward primer (10 µM), 2 µL hexachlorofluorescein (HEX) labeled reverse primer (10 µM) and bisulfite-treated DNA in a final volume of 50 µL. The PCR amplification was carried out under the following procedure: 95 °C for 15 min, and then 40 cycles of PCR at 94 °C for 30 s, 48 °C for 30 s and 72 °C for 30 s, followed by final extension for 2 min at 72 °C. After that, prechilled ethanol (1 mL, 100%) and 10 µL CH3COONa–CH3COOH buffer (3 M, pH 5.2) were added into this mixture for precipitation. This mixture was frozen at −80 °C for 30 min and then centrifuged at 4 °C (12,000× *g*) for 20 min. After removal of the supernate, the DNA precipitate was vacuum dried, then re-dissolved in ddH_2_O.

### 3.6. Preparation of G-Ladder

2 µL HEX labeled DNA (100 µM), 10 µL Tris-EDTA buffer (100 mM, pH 7.8), 86 µL distilled water and 2 µL DMS were added into the G reaction tube. The mixture was incubated at 30 °C for 8 min, followed by adding 100 µL stop buffer. The stop buffer containing 50 µL distilled water, 10 µL 3 M Na-Acetate (pH 5.2), and 40 µL ethanethiol. Then 1 mL prechilled 100% ethanol and 10 µL 3 M Na-Acetate (pH 5.2) were added into the reaction tube and vortexing. The mixture was frozen at −80 °C for 2 h and centrifuged at 4 °C (12,000× *g*) for 20 min. After removal of the upper solution, the DNA precipitate was vacuum dried, then re-dissolved in 100 µL piperidine (*v*/*v* 10%) and heated at 90 °C for 40 min. After the piperidine treatment, the DNA was precipitated again as the step mentioned above. After being dried by vacuum, the DNA precipitate was dissolved in 80% formamide deionized for further examination on acrylamide gels.

### 3.7. Photooxidation of DNA by Methylene Blue

To 5 µL 1.5 M NaCl, 0.5 M Tris-HCl solution buffer (pH 8.5), 2 µL DNA (10 µM) was added to 20 µL 0.1% methylene blue in water. The mixture was irradiated by a high pressure mercury lamp (GHg-50A) in dark at room temperature for 15 min. O_2_ was bubbled into the system during the course of the reaction. After that, the DNA was precipitated as mentioned above. After hot piperidine treatment, the DNA was precipitated as the procedures mentioned above and then dried by vacuum concentration meter.

### 3.8. RNA Digestion by RNase T1

RNA was incubated at 4 °C with RNase T1 (0.2 units) in the presence of 5 mM MgCl_2_, 200 mM NaCl and 10 mM Tris-HCl (pH 7.6). After 20 min, 1 mL prechilled ethanol (100%) and 10 µL 3 M Na-Acetate (pH 5.2) were added into this mixture. The mixture was then frozen at −80 °C for 2 h and centrifuged for 20 min. After removal of the upper solution, the RNA precipitate was vacuum dried. Finally, the RNA precipitate was dissolved in 80% formamide, deionized for further examination on acrylamide gel.

### 3.9. Enzymatic Digestion of AP-Oxidized DNA

100 µM DNA (4 µL) was treated with AP and then desalted by 3 kDa Millipore. The protocol of the completed digestion of DNA was referenced the previous report [25]. The oxidized DNA was incubated with 360 units S1 nuclease in 1 × S1 nuclease buffer at 37 °C for 16 h. Then the resulting solution was subsequently added 30 units of alkaline phosphatase and incubated in 1 × alkaline phosphatase buffer (50 mM Tris-HCl, 10 mM MgCl_2_, pH 9.0) at 37 °C for 4 h. The digested DNA was subjected to ultrafiltration tube (10 kDa cutoff, Amicon, Millipore, Shanghai, China) to remove the enzymes.

### 3.10. LC-MS Analysis of the AP-Oxidized DNA

The column temperature was set at 35 °C. Water containing 0.1% formic acid (*v*/*v*, solvent A) and methanol with 0.1% formic acid (*v*/*v*, solvent B) were employed as mobile phase with a flow rate of 0.2 mL/min. A gradient of 5% B for 5 min and 5−80% B for 20 min, last for 7 min, followed with 80%–5% B in 3 min and again last for 5 min was used.

### 3.11. Preparation of Denaturing Polyacrylamide Gel Electrophoresis (PAGE)

A 20% denaturing PAGE was prepared by using 1 × TBE buffer (89 mM Tris base, 89 mM Boric acid, 2 mM EDTA) containing 7 M urea.

## 4. Conclusions

In summary, we have proposed a novel approach for the recognition of guanine in nucleic acids by ammonium persulfate. The advantages of lower toxicity, low cost, easy repetition and wider application can make it an effective way to produce successful G sequencing in nucleic acids. Furthermore, with PCR, the detection of 5-methylcytosine successfully transformed into detection of guanine by ammonium persulfate.

## Supplementary Materials

The supplementary materisla are available online.
