# Supplementary material for: Application of Ammonium Persulfate for Selective Oxidation of Guanines for Nucleic Acid Sequencing
Source: Molecules. 2017 Jul 21;22(7):1222. doi: 10.3390/molecules22071222 (PMC6152272; doi:10.3390/molecules22071222)
Supplement: Supplementary file 1 [file molecules-22-01222-s001.pdf]

Supporting Information

Application of Ammonium Persulfate for Selective Oxidation of Guanine for Nucleic Acid Sequencing

*Yafen Wang [1†], Chaoxing Liu [1†], Tingting Hong [1], Fan Wu [1], Shuyi Yu [1], Zhiyong He [1], Wuxiang Mao [2], and Xiang Zhou[*1]*

[a]College of Chemistry and Molecular Sciences, Institute of Advanced Studies, Wuhan University, Wuhan, Hubei, 430072, P. R.China

[b]Hubei Collaborative Innovation Center for Green Transformation of Bio-resources, College of Life Sciences, Hubei University, Wuhan, Hubei 430062, P. R. China.

*To whom correspondence should be addressed:

xzhou@whu.edu.cn

List of Contents:

1. Table S1. The sequences of DNA and RNA used in this study.
2. Polyacrylamide gel electrophoresis analyzed other oligodeoxyribonucleotides.
3. Circular dichroism spectra of G-quadruplexes and G-triplex.
4. MALDI-TOF-spectrum of ODN treated with AP.

1. Table S1. DNA oligonucleotides used in this study.

Name	Sequences (5'-3')
Hairpin-loop	AGTCTATTGTTTAGACT-HEX
Bulge	ACGATCGTCATGTCTAGCAGCTA-HEX
Bulge-complementary	TAGCTGCTAGAATGACGATCGT
Loop and bulge	HEX-TCATCGATGCGTCATTGGTATGACGCGGTATCGATGA
Mismatch	ACGATCGTCATGTCTAGCAGCTA-HEX
Mismatch-complementary	TAGCTGCTAGAGATGACGATCGT
Terminal	HEX-CATGCGTTCCCGTG
Terminal -complementary	CACGGGAACGCATG
G-quadruplex	HEX-TTAGGGTTAGGGTTAGGGTTAGGG
ds	TGTGTCGCTCTTACAAGGCA-HEX
ds-complementary	TGCCTTGTAAGAGCGACACA
ZG4	TGGTGGTGGTGGTTGTGGTGGTGGTGTT-HEX
G3	HEX-TGGGTAGGGCGGG
8-oxo-G	TTTTCGATCTTACAA(8-oxo-G)CCA
Single 1	FAM-TGCGTCTGCGTC
Single 2	FAM-TCACTGTGCAGCATGTGGCAG

Single 3	HEX-CTTTGGTCCTGAAGGAGGATAGG
33mer-RNA	HEX-CUCGCAUCGAUGAAGAACGCAGCGAAAUCCCAU
22mer-RNA	HEX- CAAGCCUGUUUGACAUACAUUU
76-template-1mC	CCTCACCATCTCAACCAATATTATATTACGCGTATAA(5mC)GCGTA TTGGCGCTATAATATTGAGGGAGAAGTGGTGA
76-template-2mC	CCTCACCATCTCAACCAATATTATATTACGCGTATAA(5mC)G(5mC) GTATTGGCGCTATAATATTGAGGGAGAAGTGGTGA
76mer template-forward primer	GGGTTTTATTATTTTAATTAATATTATATT
76mer template-reverse primer	HEX-TCACCACTTCTCCCTCAAT

2. Polyacrylamide gel electrophoresis analyzed other oligodeoxyribonucleotides.

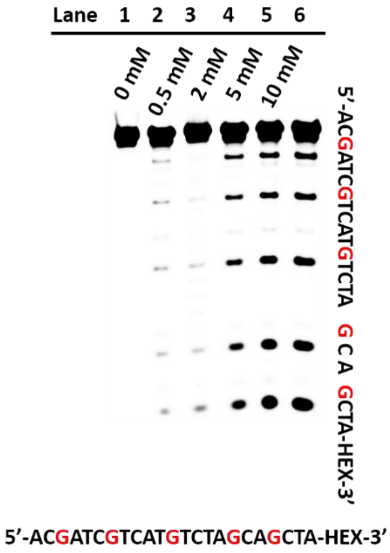

Figure S1. Polyacrylamide gel electrophoresis analysis of ODN-mismatch with AP for incubating with different concentration. Treat the DNA (20 pmol) with AP then treated with piperidine at 90 ℃ for 40 min. Lane 1-5 was 0, 0.5 mM, 2 mM, 5 mM and 10 mM respectively. Lane 6: G-ladder (DMS treated).

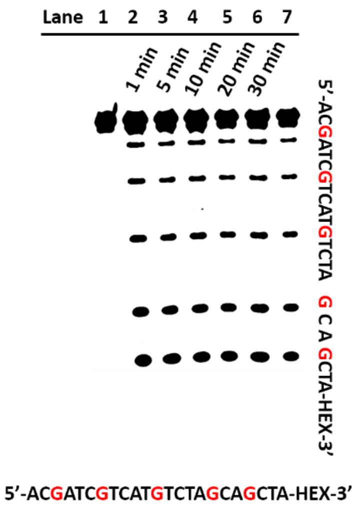

Figure S2. Polyacrylamide gel electrophoresis analysis of ODN-mismatch with AP for incubating with different time. Treat the DNA (20 pmol) with AP then treated with piperidine at 90 ℃ for 40 min. Lanes 1-6 was 0, 1 min, 5 min, 10 min, 20 min and 30 min respectively. Lane 7: G-ladder (DMS treated).

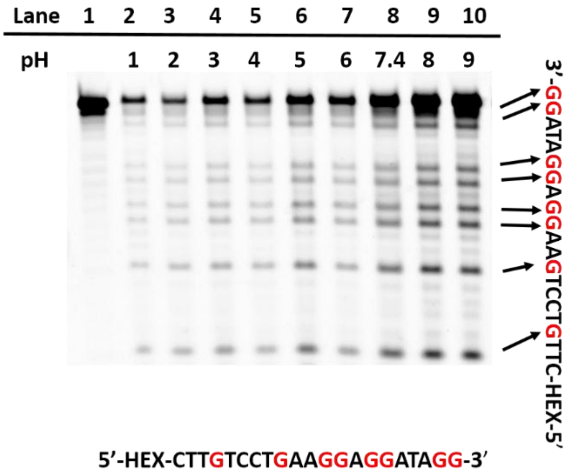

Figure S3. Polyacrylamide gel electrophoresis analysis of 21mer-ODN, which was oxidized in Tris-HCl buffer at different pH values.

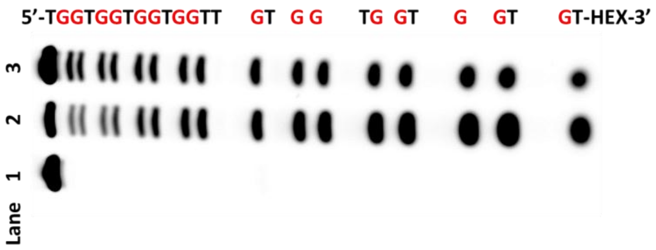

Figure S4. Polyacrylamide gel electrophoresis analysis of G-quadruplex ODN-ZG4. Lane 1: DNA without treatment; Lane 2: DNA was treated with AP; Lane 3: DNA was treated with DMS.

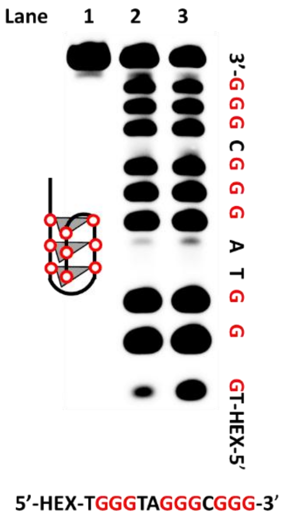

Figure S5. Polyacrylamide gel electrophoresis analysis of G-triplex structure of ODN-G3. Lane 1: DNA without treatment; lane 2: DNA was treated with AP; lane 3: DNA was treated with DMS.

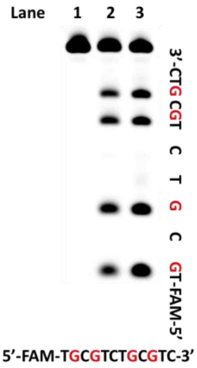

Figure S6. Polyacrylamide gel electrophoresis analysis of ODN-single 1. Lane 1: DNA without treatment; lane 2: DNA was treated with AP; lane 3: DNA was treated with DMS.

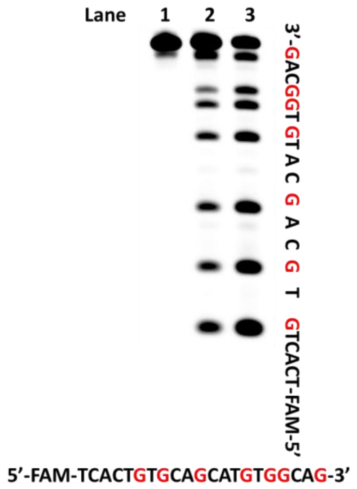

Figure S7. Polyacrylamide gel electrophoresis analysis of ODN-single 2. Lane 1: DNA without treatment; lane 2: DNA was treated with AP; lane 3: DNA was treated with DMS.

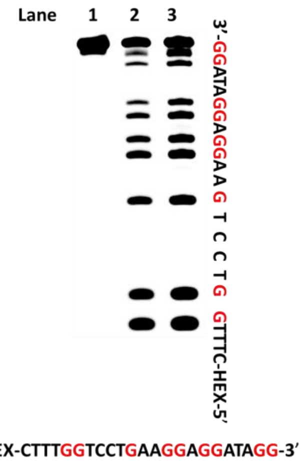

Figure S8. Polyacrylamide gel electrophoresis analysis of ODN-single 3. Lane 1: DNA without treatment; lane 2: DNA was treated with AP; lane 3: DNA was treated with DMS.

3. Circular dichroism spectra of G-quadruplexes and G-triplex.

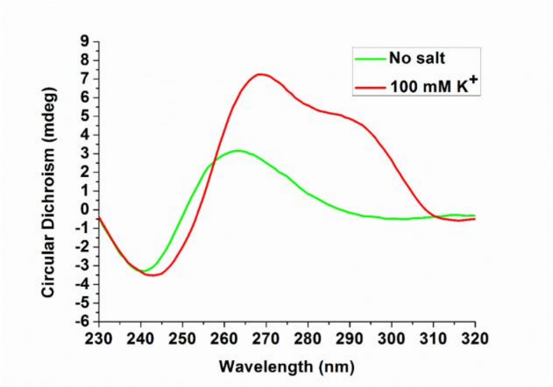

Figure S9. Spectra of 7.5 μM DNA G-quadruplex. Green line: no salt; red line: in presence of 100 mM K^+. The hybrid-type G-quadruplex showed a positive peak at 290 nm and a characteristic shoulder peak at 270 nm.

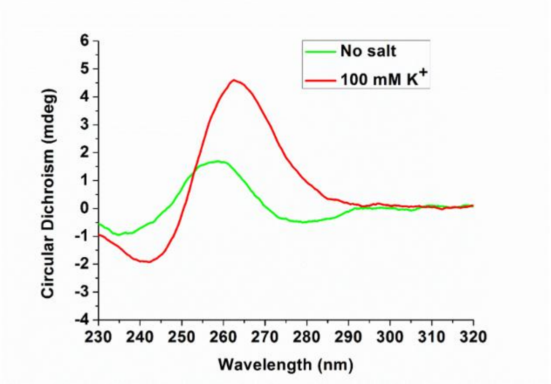

Figure S10. Spectra of 7.5 μM DNA G-triplex. Green line: no salt; red line: in presence of 100 mM K^+. The G-triplex showed a positive peak at 265 nm and a negative peak at 240 nm.

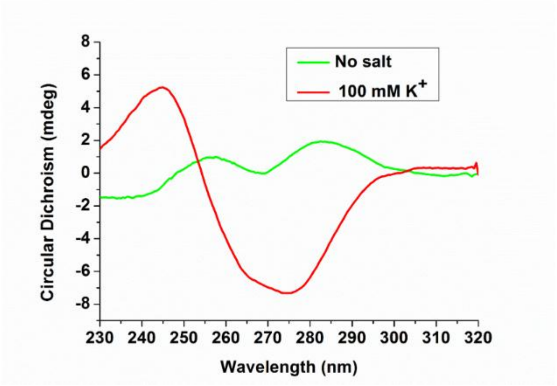

Figure S11. Spectra of 7.5 μM DNA G-quadruplex. Green line: no salt; red line: in presence of 100 mM K^+. The ZG4-quadruplex showed a positive peak at approximately 245 nm and a negative peak at 275 nm.

4. LC-MS spectrum of ODN treated with AP.

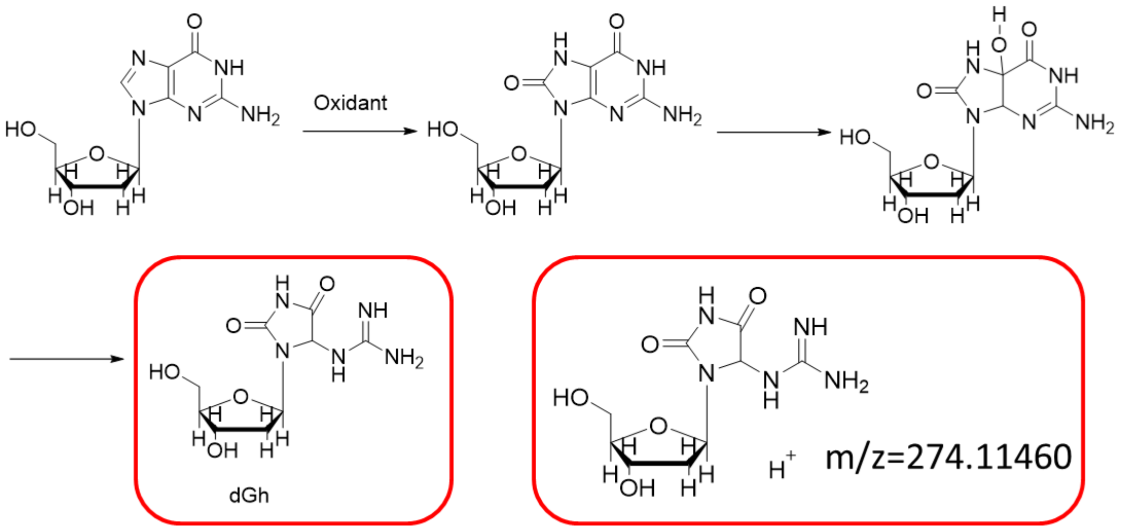

Figure S12. The guanosine could be oxidized to the labile product.

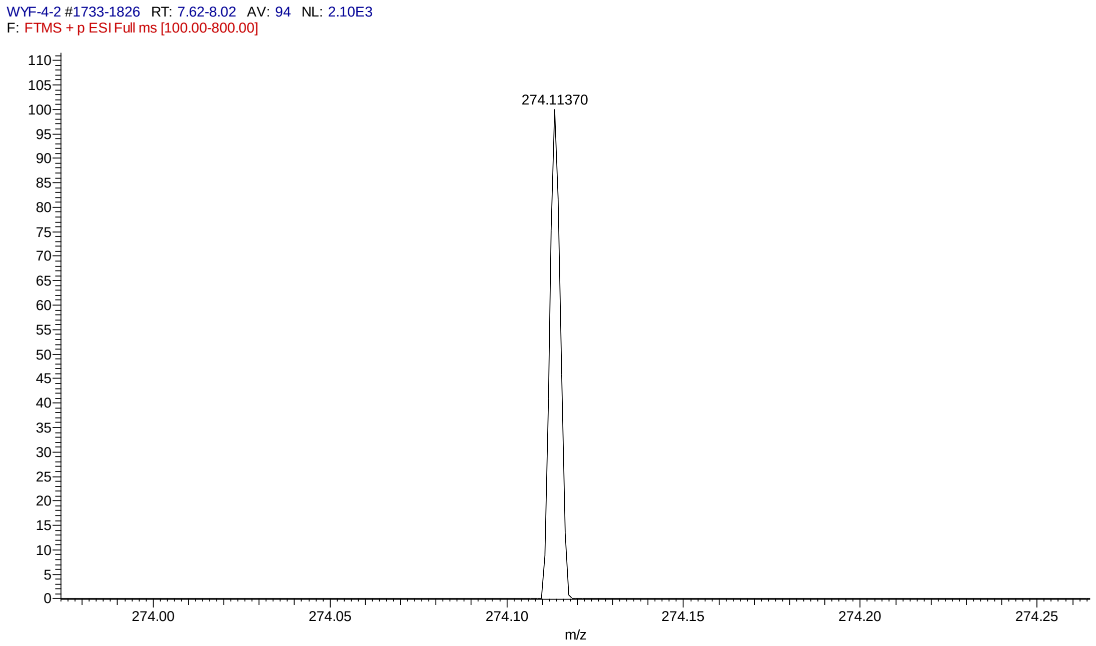

Figure S13. HPLC-MS extracted $[M+H]^+$ ion count for dGh after digestion of ODN after treatment with AP. The mass theoretical value of dGh is 274.11460, and we obtained a value of 274.11370. The sequence of the digested DNA is TAGCTGCTAGAGATGACGATCGT.
